# THE COGNITION IN PRIMARY CARE PROGRAM: A STEP-BY-STEP CLINIC WORKFLOW PUTTING THE GSA KAER TOOLKIT INTO ACTION

**DOI:** 10.1093/geroni/igad104.2956

**Published:** 2023-12-21

**Authors:** Jaqueline Raetz, Annette Fitzpatrick, Basia Belza, Sarah McKiddy, Amy Hsu, Joshua Liao, Christina Park, Barak Gaster

**Affiliations:** University of Washington, Seattle, Washington, United States; University of Washington, Seattle, Washington, United States; University of Washington, Seattle, Washington, United States; University of Washington, Seattle, Washington, United States; University of Washington, Seattle, Washington, United States; University of Washington, Seattle, Washington, United States; University of Washington, Seattle, Seattle, Washington, United States; University of Washington, Seattle, Washington, United States

## Abstract

In 2020 University of Washington (UW) Medicine began developing Cognition in Primary Care (CPC), a quality improvement program adapting the GSA KAER Toolkit to help primary care providers (PCPs) detect patients with cognitive impairment in their 16-clinic primary care network. The CPC consists of a 3-part training program to help PCPs diagnose, manage and counsel patients; a set of tools embedded into the electronic health record to facilitate cognitive evaluation; and an in-clinic workflow providing on-site support for providers within their own clinic setting. During this process we interviewed providers and developed a hybrid remote and in-person orientation for clinics to initiate a workflow to support improved cognitive evaluations. Utilizing pre-implementation input, the CPC in-clinic workflow was pilot-tested at two UW primary care clinics in 2021/2022. This presentation will describe content of the clinic orientation and implementation of CPC to include 1) the typical timeframe and content of orientation; 2) meetings with clinic leadership; 3) overview of provider/staff meetings; 4) in-person walk-through for placement of materials; 5) invitation to training; 6) a 12-page handbook outlining steps in the workflow; 7) other printed CPC materials, e.g., dementia warning signs, MoCA and AD8 forms, Alzheimer Association referral cards; and 8) follow-up focus group feedback. Evaluation of the pilot clinics following implementation found significant use of all tools by PCPs and an increase in diagnoses of mild cognitive impairment. This clinic workflow may be useful as a model for other health care systems to initiate cognitive evaluation programs in primary care.

